# Invisible Links: Associations Between Micronutrient Deficiencies and Postpartum Depression—A Systematic Review

**DOI:** 10.3390/life15101566

**Published:** 2025-10-08

**Authors:** Charalampos Voros, Ioakeim Sapantzoglou, Diamantis Athanasiou, Despoina Mavrogianni, Kyriakos Bananis, Antonia Athanasiou, Aikaterini Athanasiou, Georgios Papadimas, Charalampos Tsimpoukelis, Athanasios Gkirgkinoudis, Ioannis Papapanagiotou, Dimitrios Vaitsis, Aristotelis-Marios Koulakmanidis, Sofia Ivanidou, Anahit J. Stepanyan, Maria Anastasia Daskalaki, Nikolaos Thomakos, Marianna Theodora, Panagiotis Antsaklis, Fotios Chatzinikolaou, Dimitrios Loutradis, Georgios Daskalakis

**Affiliations:** 1 1st Department of Obstetrics and Gynecology, ‘Alexandra’ General Hospital, National and Kapodistrian University of Athens, 80 Vasilissis Sofias Avenue, 11528 Athens, Greece; kimsap1990@hotmail.com (I.S.); tsimpoukelischa@gmail.com (C.T.); aristoteliskoulak@gmail.com (A.-M.K.); md181341@students.euc.ac.cy (M.A.D.); thomakir@hotmail.com (N.T.); martheodr@gmail.com (M.T.); panosant@gmail.com (P.A.); gdaskalakis@yahoo.com (G.D.); 2 IVF Athens Reproduction Center V. Athanasiou, 15123 Maroussi, Greece; diamathan16@gmail.com (D.A.); antoathan16@gmail.com (A.A.); diamathan17@gmail.com (A.A.); 3 King’s College Hospitals NHS Foundation Trust, London SE5 9RS, UK; kyriakos.bananis@nhs.net; 4 Athens Medical School, National and Kapodistrian University of Athens, 15772 Athens, Greece; dr.georgepapadimas@gmail.com (G.P.); gpapamd@hotmail.com (I.P.); vaitsisdim@gmail.com (D.V.); info@ivanidou.gr (S.I.); anahitstepanyanj@gmail.com (A.J.S.); loutradi@otenet.gr (D.L.); 5 Laboratory of Forensic Medicine and Toxicology, School of Medicine, Aristotle University of Thessaloniki, 54124 Thessaloniki, Greece; fotischatzin@auth.gr; 6 Fertility Institute-Assisted Reproduction Unit, Paster 15, 11528 Athens, Greece

**Keywords:** postpartum depression, micronutrients, vitamin D, vitamin B12, folic acid, iron, zinc, magnesium, selenium, maternal health, nutrition, prenatal, systematic review

## Abstract

Background: Following childbirth, up to 20% of women may have postpartum depression (PPD), which can adversely affect the mother’s health, the infant’s development, and familial connections. Numerous causes exist, although recent research indicates that micronutrient shortages are modifiable biological factors. This systematic review aims to consolidate existing knowledge regarding the relationship between micronutrient levels and the risk of PPD. Methods: This review was conducted in accordance with PRISMA 2020 guidelines and registered with PROSPERO. We reviewed every study published up to 1 April 2025, on PubMed, Scopus, and Web of Science. Nineteen studies met the inclusion criteria. We employed the Newcastle–Ottawa Scale to assess bias. Results: Nineteen studies were included in the analysis. Vitamin D was the most extensively researched vitamin. The majority of the studies (9 out of 13) identified a significant correlation between low serum 25(OH)D levels and PPD symptoms. Individuals with diminished levels of vitamin B12 and zinc had an elevated risk of PPD. There was insufficient evidence for folate, magnesium, iron, and selenium. This was frequently due to methodological discrepancies, insufficient control of confounding variables, and variations in biomarker timing. The majority of the studies exhibit a low to moderate likelihood of bias. Conclusions: Increasing evidence suggests that deficiencies in specific micronutrients, particularly vitamin D, vitamin B12, and zinc, may contribute to the onset of postpartum depression. The results indicate that targeted nutritional screening and management may be beneficial in perinatal mental health care, notwithstanding the inability to ascertain the exact causative factors. There is a necessity for more rigorous longitudinal investigations and randomised trials to enhance our understanding of processes and assist physicians in making informed judgements.

## 1. Introduction

### 1.1. Definition and Prevalence of Postpartum Depression (PPD)

PPD is currently recognised as one of the most prevalent outcomes of the perinatal period, constituting a serious and complex mood disorder that affects women after childbirth [1]. The Diagnostic and Statistical Manual of Mental Disorders, Fifth Edition (DSM-5), categorises PPD as a subtype of major depressive disorder with peripartum onset, applicable when symptoms emerge during pregnancy or within four weeks postpartum [2]. Due to the persistence and delayed onset of depressive symptoms in numerous affected individuals, many experts and studies extend the diagnostic window to encompass the entire first year postpartum [3].

The amalgamation of emotional, cognitive, behavioural, and physical symptoms exhibited by PPD significantly undermines a mother’s functionality and quality of life [4]. PPD causes a lot of physical and mental problems that make it hard for a mother to do her job [5,6].

Postpartum depression (PPD) is prevalent globally and is a significant public health concern. Although the true prevalence may be exacerbated by underdiagnosis and societal stigma surrounding maternal mental health, epidemiological studies indicate that 10–20% of women worldwide experience postpartum depression (PPD) [7]. The geographic area, sociodemographic context, utilised screening methods, and diagnostic thresholds significantly influence prevalence. Research utilising the EPDS, a validated self-report instrument, indicates prevalence rates between 6% and 25%, with elevated rates observed in low- and middle-income countries (LMICs) where systemic barriers to care and nutritional insecurity are prevalent [8]. Rates in certain high-risk populations, like adolescent mothers, individuals with a history of mood disorders, and those experiencing intimate partner abuse, may exceed 30% [9]. PPD has bad effects on the child’s growth and the family’s relationships, not just the mother’s [1,10,11].

Clinically, numerous instances remain undetected despite the existence of screening instruments such as the EPDS and the Centre for Epidemiologic Studies Depression Scale (CES-D), especially among women who are reluctant to disclose symptoms due to concerns about judgement, stigma, or the potential loss of parental rights. Furthermore, inadequate incorporation of prenatal mental health into standard obstetric care results in treatment deficiencies, even within well-resourced healthcare systems.

While psychological and hormonal factors are evidently implicated in the onset and severity of PPD, recent studies suggest that biological processes such as inflammation, oxidative stress, and nutritional deficiencies may significantly contribute to the condition’s development [12]. Maternal micronutrient levels represent a significant modifiable biological factor; their potential impact on neurochemical imbalances, immune activation, and altered neuroendocrine signalling associated with postpartum depression is increasingly being investigated [13].

### 1.2. Genetic, Psychological, and Biological Aspects Define the Risk Factors for PPD

Considered as the outcome of a complex interaction of psychosocial, genetic, hormonal, and biological factors, PPD is somewhat common. Development of preventative plans and guidance of clinical screening procedures depend on the identification and categorisation of these risk factors. Key PPD risk categories—including psychosocial factors, psychiatric history, maternal problems, neuroendocrine mechanisms, immune-inflammatory responses, and dietary inadequacies—are discussed in this part. The risk of PPD has been repeatedly linked to many psychosocial variables, including insufficient social support, marital conflict, unwanted pregnancy, socioeconomic deprivation, teenage parenthood, adverse life experiences, and minority status [14,15,16,17,18,19,20,21,22,23,24,25,26,27].

#### 1.2.1. Historical Overview of Mental Illness and Personal Life

A prior personal or familial history of psychiatric disorders is one of the most significant and commonly documented predictors of PPD. Women with histories of major depressive disorder (MDD), generalised anxiety disorder (GAD), bipolar disorder (BD), or premenstrual dysphoric disorder (PMDD) represent a high-risk subgroup, with their vulnerability potentially intensified during the perinatal period due to neuroendocrine, immune, and psychosocial stressors. The probability of PPD in these demographics is significantly elevated, with meta-analyses revealing odds ratios (ORs) ranging from 3.0 to 6.0 for those with a previous history of affective disorders [28].

The transition to parenthood involves substantial neurobiological and psychosocial alterations that may act as triggers for relapse in women with a history of mood instability. Women with a history of MDD are especially susceptible to recurrent depressive episodes during or during pregnancy, particularly if the previous episodes were hormonally sensitive or triggered by stress [29]. Bipolar disorder, especially bipolar II disorder, carries a considerable risk of postpartum affective episodes, including both depressive and hypomanic states [30]. The misdiagnosis or underdiagnosis of BD during the perinatal period is common, as depressive symptoms may be mistakenly attributed to unipolar depression, leading to inadequate treatment and an increased risk of rapid cycling or psychosis [31].

Antenatal mood disorders are a critical and often overlooked predictor of PPD. Antenatal depression (AND) and antenatal anxiety (ANA) are common symptoms associated with pregnancy and can persist if not properly managed [32]. Longitudinal studies indicate that 50–70% of women with antenatal depression (AND) may subsequently experience postpartum PPD, underscoring the persistence of mood disorders during the perinatal period [33]. Symptoms such as persistent sadness, anhedonia, excessive worry, irritability, physical ailments (including fatigue and abdominal discomfort), and sleep disturbances throughout pregnancy are significant indicators that a woman may experience emotional difficulties postpartum [34,35].

AND is frequently underappreciated in clinical environments, as prenatal healthcare appointments predominantly stress the physical well-being of the mother and foetus, sometimes neglecting the mother’s mental health. Cultural stigma, fear of judgement, and inadequate awareness of pregnant mental illness may lead women to conceal their symptoms [36]. As a result, prospects for early intervention are frequently neglected. Moreover, untreated ANA has been linked to PPD as well as adverse obstetric outcomes, including premature birth, low birth weight, and inadequate bonding, highlighting the imperative for routine screening and comprehensive therapy approaches.

The concurrent occurrence of anxiety and depressive symptoms, referred to as mixed anxiety–depressive disease, is particularly relevant in the context of PPD. Women with heightened trait anxiety or excessive worry regarding their pregnancy, childbirth, or parenting competence are at a greater risk of experiencing persistent depression symptoms after childbirth [37]. Elevated anxiety during pregnancy may arise from concerns of labour pain, miscarriage, foetal abnormalities, or a perceived lack of control and is often exacerbated in first-time mothers, persons with a history of infertility, or those undergoing medically complex pregnancies [38].

A personal or family history of mental illness, antenatal mood disorders, trauma, or maladaptive coping mechanisms significantly heighten vulnerability to PPD [27,39].

Familial history is a crucial determinant in assessing the risk of PPD. First-degree relatives with a history of MDD, BD, or prenatal affective disorders increase the genetic and epigenetic susceptibility of the individual, likely through alterations in serotonergic, dopaminergic, and HPA axis pathways. Family history is rarely assessed in normal prenatal care, yet it can yield crucial insights into baseline vulnerability and dictate the intensity of postpartum monitoring.

#### 1.2.2. Reproductive and Obstetric Considerations

For a subgroup of women, obstetric and reproductive factors, while not as reliably predictive as psychological or psychosocial variables, significantly impact the onset of PPD. Emotional outcomes during the postpartum period encompass the mode of delivery, perceived birth experience, complications during labour, and early neonatal difficulties.

PPD risk has been associated with obstetric complications, caesarean delivery, perinatal loss, NICU admission, and stress related to parity [40,41,42,43,44]. Pregnancy and delivery experiences can both increase and alter the risk of postpartum depression. While not all women experiencing challenging births or adverse neonatal outcomes develop mood disorders, those with additional psychosocial or psychiatric vulnerabilities appear more susceptible. Timely identification and intervention rely on the incorporation of mental health assessments into obstetric care, especially in instances of delivery complications or NICU admission.

#### 1.2.3. Hormonal and Neuroendocrine Factors

In susceptible women, sudden postpartum hormonal fluctuations (including oxytocin, progesterone, cortisol, thyroid hormones, prolactin, and oestrogen) engage with stress pathways and neurotransmitter systems, resulting in PPD [45,46,47,48,49,50,51,52]. Taken together, hormonal and neuroendocrine variables comprise a major physiologic axis in PPD pathogenesis. Their molecular relevance is still under investigation, although their association with mood-regulating systems underscores the importance of early identification of endocrine anomalies, particularly in individuals at high risk with a history of reproductive mood disorders or hormone sensitivity. The aetiology of PPD involves immunological dysregulation and inflammation, including changes in cytokines, activation of the kynurenine pathway, and gut–brain connections [53,54,55,56,57,58,59].

### 1.3. The Impact of Micronutrients on Mood

Micronutrients are crucial for regulating mood, cognition, neuroendocrine signalling, mitochondrial function, and immune homeostasis. Their roles are particularly crucial during the postpartum phase, when the mother’s metabolic requirements increase and her bodily reserves may be depleted. A deficiency in several micronutrients, including iron, zinc, magnesium, vitamin D, vitamin B12, folate, and selenium, has been repeatedly associated with an increased risk of affective disorders, such as postpartum depression [60]. These micronutrients function as enzyme cofactors, hormone regulators, and modulators of oxidative stress, significantly influencing neurochemical equilibrium and neuroplasticity.

Iron is essential for oxygen transfer, cellular respiration, and the synthesis of dopamine, norepinephrine, and serotonin. Iron is involved in myelination and mitochondrial function in the central nervous system, and it restricts the activity of tyrosine and tryptophan hydroxylase [61]. Postpartum iron insufficiency, typically resulting from intrapartum haemorrhage, excessive erythropoiesis, or inadequate dietary intake, may reduce energy metabolism in neuronal cells and disrupt monoaminergic signalling. Research suggests that reduced ferritin levels during the postpartum period are associated with impaired cognitive performance, fatigue, apathy, and depressive symptoms. Isolated low ferritin levels, even without anaemia, can correlate with an increased risk of PPD, indicating that iron storage, rather than solely haemoglobin concentration, plays a critical role [62].

Zn is a crucial regulator of synaptic plasticity, neurogenesis, and the activity of more than 300 Zn-dependent enzymes. It regulates glutamatergic and GABAergic neurotransmission, maintains neuronal membrane stability, and functions as an antioxidant by inhibiting NADPH oxidase and modulating metallothioneins [63]. Insufficient zinc increases the activity of NMDA receptors, leading to excitotoxicity that harms the hippocampus and heightens the likelihood of mood fluctuations. Reduced serum zinc concentrations have consistently been associated with depressive symptoms in both pregnant and postpartum women. Zinc influences the functionality of the HPA axis and the body’s cortisol response, indicating that insufficient levels may exacerbate stress-induced neuroinflammation and contribute to HPA dysregulation in PPD [64].

Over 600 enzymatic reactions rely on magnesium, many of which are crucial for central nervous system excitability, synaptic integrity, and mitochondrial function. It functions as a natural calcium antagonist and NMDA receptor inhibitor, reducing excitotoxicity and safeguarding neurons. A deficiency in magnesium leads to increased production of CRH by the brain, inhibits GABAergic activity, and heightens the brain’s sensitivity to stressors. In perinatal women, diminished magnesium levels are associated with increased anxiety, insomnia, muscle tension, and depressive symptoms [65,66]. Magnesium may serve as a mechanistic link between biological and psychological processes contributing to postpartum PPD due to its effects on neuromodulation and systemic inflammation.

Vitamin D is increasingly recognised as a neurosteroid that influences various aspects of cerebral function. It traverses the blood–brain barrier, binds to nuclear receptors in the hippocampus, prefrontal cortex, and amygdala, and alters the expression of genes associated with neurotrophin synthesis, calcium homeostasis, and oxidative stress protection. Vitamin D regulates TPH2, influencing the synthesis of 5-HT in the brain, while also inhibiting pro-inflammatory cytokines such as IL-6 and TNF-α. Numerous observational studies have demonstrated that women with postpartum depression exhibit reduced levels of 25(OH)D, even when controlling for seasonality, BMI, and other potential confounding variables. Deficiency is more prevalent in individuals at elevated risk, including those with limited sun exposure, darker skin pigmentation, or dietary restrictions [67,68].

Vitamin B12 and folate are essential for one-carbon metabolism, methylation activities, and the synthesis of S-adenosylmethionine (SAM), a crucial methyl donor for neurotransmitter and phospholipid metabolism. Insufficient levels of B12 or folate can elevate homocysteine concentrations, which adversely affect the brain and are associated with vascular damage, oxidative stress, and impairments in neurogenesis [69]. Elevated homocysteine levels have been observed in individuals with severe depression, and they are also elevated in certain instances of PPD. Polymorphisms in MTHFR, such as C677T, can disrupt folate metabolism despite adequate intake, hence increasing the biological risk of depression in genetically predisposed individuals [70].

Selenium improves antioxidant capacity through its incorporation into selenoproteins such as GPx, which neutralizes hydrogen peroxide and protects neuronal membranes from oxidative damage. It possesses anti-inflammatory effects and alters the functioning of the HPA axis. Observational studies suggest that women with insufficient selenium intake or serum levels are at a higher risk of experiencing depressive symptoms during the postpartum period. Despite the limited availability of interventional trials, some evidence suggests that selenium supplementation during pregnancy may reduce the incidence and intensity of PPD symptoms [71,72].

These micronutrients function synergistically, rather than in isolation, which is significant. Numerous deficiencies frequently coexist, particularly in resource-constrained settings or among women with restricted diets (e.g., vegetarianism, food insecurity, or eating disorders). A deficiency in one nutrient might exacerbate the effects of another. For instance, insufficient magnesium might impede the efficacy of vitamin D, while a deficiency in B12 can hinder the utilisation of folate. Furthermore, inflammation can diminish serum concentrations of Fe, Zn, and Se by sequestering and redistributing these elements via cytokines. This establishes a feed-forward cycle that exacerbates nutritional and mood-related issues.

### 1.4. The Imperative for a Methodical Review of the Literature

Despite the growing body of evidence linking micronutrient deficiencies to PPD, the current literature remains inconsistent, fragmented, and methodologically varied. Observational studies, clinical trials, and meta-analyses have yielded substantial insights; however, the lack of standardization in study design, micronutrient assessment, diagnostic criteria, and outcome measurement limits the generalizability and translational applicability of existing findings.

The heterogeneity of research populations leads to considerable differences in dietary status, genetic predisposition, socioeconomic level, and prenatal care practices. These factors affect the prevalence of micronutrient deficiencies and the expression of depressive symptoms; nevertheless, they are often underreported or insufficiently controlled in primary studies. Furthermore, studies differ in their assessment of dietary intake, blood biomarkers, or supplementation records, each potentially reflecting unique facets of micronutrient status and bioavailability.

The diagnostic criteria for PPD vary among studies; some utilize structured psychiatric interviews, while others use self-report tools such as the EPDS, PHQ-9, or BDI. The procedures differ in sensitivity, specificity, and timing of postpartum testing, resulting in inconsistent prevalence estimates and difficulties in comparing results. Moreover, scant research clearly distinguishes between early-onset depressive symptoms and later signs of mood disorders or examines the evolution of symptoms during the postpartum year [73].

Third, whereas individual micronutrients—such as iron, zinc, or vitamin D—have been thoroughly investigated, there is a scarcity of studies exploring their synergistic or cumulative effects. Deficiencies in micronutrients rarely occur in isolation; the interplay of reduced levels of many nutrients might result in cumulative or even synergistic effects on neurobiological systems linked to PPD. Nonetheless, the majority of contemporary research uses a reductionist, single-nutrient approach, overlooking the holistic dietary context that could influence postpartum risk.

Moreover, most randomized controlled trials examining micronutrient supplementation in the postpartum period are marked by limited sample sizes, brief durations, or a concentration solely on people with specific nutritional deficiencies. The heterogeneity in dosage, duration, formulation, and outcome measures complicates the capacity to draw solid conclusions regarding efficacy. A lack of prospective cohort studies with repeated biological sampling exists, which are crucial for determining temporal relationships and causality between dietary levels and depressive symptoms.

Given these limitations, a thorough synthesis of the existing research is essential. This approach facilitates the rigorous assessment of study quality, the identification of recurring patterns or evidence deficiencies, and the development of hypotheses for future research. It facilitates the comparison of outcomes across various micronutrients, geographic regions, and research approaches. This may elucidate prevalent pathways or hazards particular to distinct demographics. A thorough study can substantially improve clinical guidelines by clarifying the deficiencies most associated with PPD and evaluating the rationale for implementing screening or supplementary interventions in normal postpartum care.

Considering the substantial prevalence of PPD, the modifiability of nutritional status, and the safety and affordability of micronutrient therapy, it is imperative to formulate evidence-based conclusions. A comprehensive and methodologically rigorous synthesis is essential to further the study, align biological plausibility with clinical applicability, and eventually assist in the prevention and early management of PPD.

### 1.5. Objective of the Present Systematic Review

This systematic review aims to meticulously evaluate and synthesize existing scientific evidence on the relationship between micronutrient deficiencies and PPD. PPD is becoming recognized as a condition with multifaceted roots, including biological, psychological, and social factors, with nutritional status emerging as an important and changeable contributor. This review focuses on deficiencies in iron Fe, Zn, Mg, Vit D, vitamin B12, folate, and selenium (Se), all of which exhibit credible mechanistic links to mood regulation through their effects on neurotransmission, neuroinflammation, and neuroendocrine function.

This review aims to systematically identify and analyze peer-reviewed studies to determine which micronutrients are most consistently associated with an increased risk or severity of PPD while also assessing the methodological quality, population characteristics, and consistency of the reported findings. Significant attention is directed towards geographical, cultural, and assessment tool discrepancies, accompanied by proposed biological mechanisms that may elucidate these correlations. The study examines the sufficiency of evidence for the implementation of micronutrient screening or supplementation in preventive or therapeutic approaches within perinatal mental health care.

## 2. Materials and Methods

### 2.1. Regulations and Enrolment

This systematic review adhered to the 2020 guidelines of the Preferred Reporting Items for Systematic Reviews and Meta-Analyses (PRISMA). The protocol was proactively filed in the PROSPERO international prospective register of systematic reviews (registration ID: CRD420251028514). The review adhered to a defined methodology that outlined the study topic, participant eligibility criteria, search strategy, data collection methodologies, and bias risk assessment.

### 2.2. Eligibility Criteria

The PICOS framework was employed to establish the qualifying criteria for this systematic review. This ensured that the study selection process was systematic and transparent. Studies considered for inclusion must investigate the relationship between micronutrient status and the incidence or severity of PPD, with well defined criteria for demographics, exposure, outcomes, and study methodology.

The intended audience was women who were either pregnant or within the first 12 months postpartum. Only studies that assessed depressive symptoms in pregnant women during the postpartum period were included, enabling prospective correlations between antenatal micronutrient status and future emotional outcomes. Research focussing only on postpartum persons was considered appropriate, regardless of parity, delivery style, or comorbidities, provided that PPD was assessed using validated instruments.

The focus of interest was the assessment of at least one micronutrient relevant to mood regulation—specifically iron, zinc, magnesium, vitamin D, vitamin B12, folate, or selenium. Eligible studies were those that evaluated micronutrient levels through biochemical assays (e.g., serum, plasma, whole blood, or RBCs), food intake methods (e.g., FFQs, 24-h recall) or recorded supplemental exposure (e.g., prenatal or postpartum micronutrient consumption). Studies assessing multiple micronutrients were retained if unique relationships with PPD were established for each.

The outcome involved the identification, diagnosis, or intensity of PPD, assessed via standardised and validated tools such as the EPDS, CES-D, BDI, or PHQ-9 or through structured psychiatric interviews according to DSM or ICD standards (American Psychiatric Association, 2013; World Health Organization, 2019) [74,75]. Studies were deemed suitable if they provided continuous or categorical postpartum depression outcomes and if the evaluation of depressive symptoms occurred within 12 months following childbirth. Studies that evaluated depression exclusively during pregnancy or did not specify the postpartum period were excluded.

Eligible study designs encompassed observational studies (cross-sectional, case–control, prospective, and retrospective cohorts) and interventional studies (RCTs or quasi-experimental trials) that provided original data. The sample size, recruiting environment (community versus hospital-based), and geographic area were not constrained. The study included high-income, low-income, and middle-income countries to illustrate global inequalities in micronutrient availability and the incidence of PPD.

The exclusion criteria comprised (i) non-human or in vitro studies; (ii) narrative reviews, meta-analyses, editorials, opinion pieces, or case reports; (iii) studies devoid of both micronutrient and PPD data; (iv) studies utilising unvalidated or non-standardized instruments for evaluating depressive symptoms; and (v) publications not available in English without a feasible translation. We excluded papers published solely as abstracts lacking comprehensive peer-reviewed data.

### 2.3. Information Sources

A systematic search was conducted across three principal electronic databases—PubMed, Scopus, and Web of Science—to comprehensively identify the pertinent literature examining the association between micronutrient deficiencies and PPD. We selected these databases because of their extensive collection of peer-reviewed literature on biomedical sciences, nutrition, psychology, obstetrics, and epidemiology. This enhances the likelihood of identifying both clinical and mechanistic research pertinent to the review subject.

A comprehensive examination of Web of Science, PubMed, and Scopus was performed, including research from the inception of the database up to 1 April 2025. This approach facilitated the incorporation of both earlier fundamental research and recent, high-caliber data demonstrating advancements in biochemical examination and psychiatric screening. We queried each database utilising precise combinations of keywords and controlled vocabulary terms (such as MeSH) related to “postpartum depression”, “micronutrients”, and particular nutrients of interest (Fe, Zn, Mg, Vit D, B12, folate, Se), in addition to prevalent depression screening instruments (such as EPDS, CES-D, BDI).

In addition to the electronic search, the reference lists of all included full-text publications were manually examined to identify further research that may have been overlooked in the database searches. This backward citation tracking proved particularly beneficial for older research or papers published in specific domains that may not be comprehensively indexed in the databases we utilised.

Only research published in English-language, peer-reviewed journals was eligible. Studies in other languages were excluded unless an English-language version or official translation was available. Grey literature, such as unpublished theses, dissertations, government reports, and conference abstracts lacking full-text publications, was excluded to maintain the methodological transparency and data quality crucial for systematic review synthesis.

The subsequent section has comprehensive details regarding the search strategies employed for each database, including specific Boolean operators, constraints, and filters.

### 2.4. Search Strategy

A systematic and reproducible search strategy was developed and executed to identify all eligible papers examining the relationship between micronutrient deficiencies and PPD. The computerised search employed three major biomedical databases—PubMed, Scopus, and Web of Science—each offering extensive indexing of peer-reviewed literature in obstetrics, psychiatry, nutrition, and public health. The final search was conducted on 1 April 2025, encompassing all records available from inception to that date. No restrictions were imposed on the year of publication to ensure the inclusion of both older and contemporary research regarding the impact of nutrition on mental health during the postpartum period.

The search strategy was developed to identify the major concepts of the review, which included the target condition PPD, the exposures of interest (Fe, Zn, Mg, Vit D, B12, folate, Se), and the validated outcome measures employed to assess depressive symptoms. We employed a combination of free-text expressions and controlled terminology (such as MeSH in PubMed), adapting them to conform to the syntax of each database. The terms employed for the condition of interest include “postpartum depression,” “postnatal depression,” “maternal depression,” and “perinatal depression.” The terms associated with exposure encompassed “micronutrients,” “nutritional deficiency,” “trace elements,” “vitamin D,” “iron,” “zinc,” “magnesium,” “vitamin B12,” “folic acid,” “folate,” and “selenium”. The search incorporated phrases such as “EPDS,” “CES-D,” “BDI,” “PHQ-9,” “depression scale,” and “depressive symptoms” to ensure the identification of studies utilising validated psychometric instruments.

We utilised Boolean operators (AND/OR) to construct intricate search strings that may link or augment pertinent terms according to their application in the query. We applied criteria to restrict the search to English-language papers, peer-reviewed journals, and items concerning individuals. We excluded records that were not published in English or were solely available as conference abstracts or editorials lacking full-text data. To ensure compliance with the indexing and search limitations of each database, we utilised the advanced search interface for every database.

We utilised Zotero (v6.0.42) reference management software to export and consolidate the results from each database. Zotero’s integrated de-duplication function automatically identified and removed duplicate items. Upon eliminating duplicates, the distinct records were prepared for the title and abstract screening phase.

In addition to the primary electronic search, manual backward citation tracking was conducted by examining the reference lists of all full-text papers included in the study. This phase facilitated the identification of additional eligible studies that were not uncovered in the initial search due to variations in indexing or delays in database entry.

### 2.5. Selection Criteria

After a systematic search of PubMed, Scopus, and Web of Science, duplicate entries were removed using Zotero (v6.0.42), and the remaining records underwent a two-stage screening process to ensure strict adherence to the set qualifying criteria. Two reviewers independently conducted the screening using a predetermined, uniform technique designed to minimise selection bias and ensure methodological rigour.

During the preliminary stage, the titles and abstracts of all obtained references were examined to exclude studies that were clearly irrelevant based on population, exposure, outcome, or research methodology. Records were excluded if they: (i) did not involve human participants during the pregnancy or postpartum period; (ii) did not provide micronutrient assessment data (biochemical, dietary, or supplemental); (iii) did not assess PPD using validated screening instruments or diagnostic interviews; or (iv) were review articles, editorials, conference proceedings, or case reports devoid of primary data. Studies considered potentially relevant or unclear were retained for thorough text analysis, ensuring a rigorous and comprehensive screening process.

During the second phase, both reviewers independently accessed and assessed the complete articles of all potentially qualifying studies. The reviewers applied identical eligibility criteria at the full-text level to determine inclusion in the final list. Any discrepancies identified during the full-text examination were resolved through discussion until consensus was reached. In the absence of consensus, a third reviewer (Δ.A.) was solicited to render the final decision about the inclusion of the work. This technique was conducted in a blinded manner regarding authorship, journal name, and institution to mitigate bias in the decision-making process.

**Figure 1:**
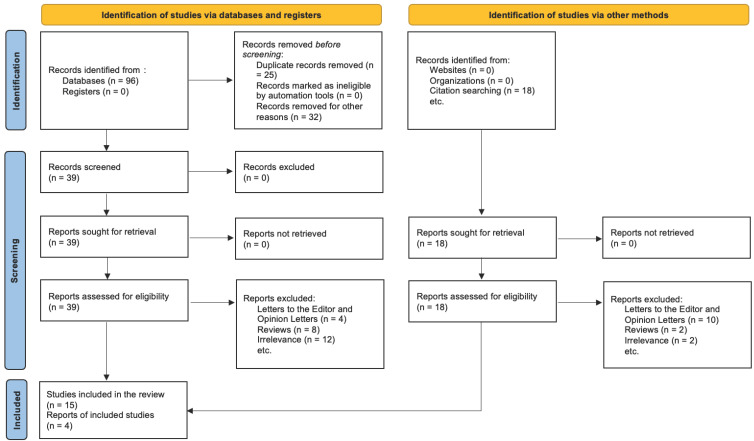
PRISMA 2020 flow diagram depicting the methodology for study identification, screening, eligibility evaluation, and inclusion in the current systematic review.

For studies excluded at the full-text stage, the precise rationale for exclusion was meticulously recorded (e.g., PPD not assessed, micronutrient unspecified, utilisation of non-validated depression scales, ineligible study design). The PRISMA 2020 flow diagram (Figure 1) distinctly illustrates the entire selection process. It indicates the number of records identified, duplicates, screened records, complete texts evaluated, exclusions with justifications, and studies ultimately incorporated into the qualitative synthesis. This rigorous, multi-phase screening procedure was implemented to ensure that only methodologically robust papers pertinent to the research question were incorporated into the systematic review.

### 2.6. Data Collection Methods

The data collection procedure followed a rigorous and standardized methodology to ensure the quality, reliability, and transparency of all collected information. Upon finalizing the selection of studies that satisfied the eligibility criteria, data were systematically extracted utilising a preconfigured and pilot-tested Excel form developed in alignment with PRISMA 2020 guidelines and the established PROSPERO protocol (CRD420251028514). This form underwent continuous enhancement until it encompassed all requisite domains for organised synthesis, including comprehensive biochemical, methodological, and clinical information.

We examined each eligible study comprehensively and extracted pertinent data across five primary domains: (i) study-level descriptors, (ii) population and clinical characteristics, (iii) exposure details concerning micronutrient measurement, (iv) PPD outcome assessment, and (v) principal results and statistical interpretations.

The primary study parameters included the first author’s surname, publication year, geographical setting, study type (e.g., cross-sectional, case–control, cohort, RCT), and publication language. Data regarding recruitment sources (such as hospitals, community centres, and online surveys), ethical approval, and financial disclosures was documented when feasible to provide context regarding the study’s origins and potential biases.

Population-level data included total sample size, participant eligibility criteria, mean or median maternal age, recruitment timing (gestational or postpartum), and relevant obstetric information, such as parity, mode of delivery, pregnancy complications, and breastfeeding status. Studies were further classified based on the enrollment of women either during the antenatal period, postpartum, or both, as well as the collection and reporting of socioeconomic, nutritional, or psychosocial characteristics.

In the exposure domain, each study was examined for the types and amounts of micronutrients assessed, with particular focus on Fe, Zn, Mg, Vit D, B12, folate, and Se. We documented the biological matrices employed for micronutrient measurement (such as serum, plasma, red blood cells, and urine) and the specific laboratory methodologies applied (including immunoassay, high-performance liquid chromatography, and atomic absorption spectroscopy). The dietary instruments employed to assess micronutrient consumption specified the format (such as FFQs, 24-h recall, or food records) and if these tools had been validated for the population under investigation. The timing of the exposure assessment concerning delivery (e.g., third trimester, 4–8 weeks postpartum) was documented to enable accurate temporal association with depressive results.

Validated instruments such as the EPDS, CES-D, BDI, and PHQ-9 were employed for PPD outcome data, with established cut-off values to ascertain the presence and severity of cases. The timing of instrument administration was recorded (e.g., at 6 weeks, 3 months, or 6 months postpartum) along with the method of administration, whether self-administered or conducted via interview. Research employing diagnostic interviews based on DSM or ICD classifications was likewise identified. If longitudinal follow-up was conducted, data on repeated measurements or trajectory modelling was included.

In Section 3, all established correlations between micronutrient status and PPD were meticulously examined. This included the direction and amount of effects, statistical significance (e.g., *p*-values, 95% confidence intervals), and the degree to which the analyses were controlled for potential confounding variables. Multivariate regression models documented the number and features of covariates, including SES, educational attainment, BMI, history of depression, parity, and breastfeeding. We also observed research that examined subgroups, such as those analyzing dietary tertiles, trimesters, or postpartum depression severity. Whenever feasible, effect sizes such as ORs, β coefficients, and RRs were directly utilized, maintaining consistent units and reference ranges for clarity.

In the absence of crucial information or clarity, we endeavored to elucidate it through the utilization of tables, figures, or footnotes. We incorporated research that presented data in narrative or descriptive formats, designating these qualitative findings for distinct synthesis to clarify the strength of the evidence.

We meticulously scrutinized the data we received on two occasions to ensure its accuracy and consistency. The finalized dataset was structured into summary tables to enable direct comparison of studies and to support the ensuing narrative synthesis. Table 1 serves as the principal descriptive matrix for the review. It provides a comprehensive overview of the attributes of each included study, including the population, exposure, outcomes, and principal findings.

### 2.7. Data Elements

The data items obtained from each included study were predetermined based on the conceptual framework of the systematic review, which sought to examine the potential correlation between particular micronutrient deficiencies and PPD. The objective of the data extraction was to ensure a comprehensive and uniform set of variables across studies, facilitating a systematic and significant synthesis of results. The data collection concentrated on five principal domains: study-level descriptors, demographic factors, micronutrient assessment parameters, PPD assessment methods, and key outcomes.

The first domain encompassed general research descriptors, including the surname of the primary author, the year of publication, and the country or region of the study’s execution. This information was employed to assess the regional distribution and temporal trends in research activities. The study design was retrieved and classified as cross-sectional, case–control, cohort, or randomised controlled trial, as the design type influences bias risk, temporal inference, and total evidence strength.

The second domain pertained to population-level characteristics. The overall sample size and the demographic characteristics of the sample (e.g., mean or median maternal age, standard deviation, age range) were documented. Criteria for participant inclusion and exclusion were collected to assess selection bias and generalisability to the population. Data about the moment of recruitment (antenatal, immediate postpartum, or late postpartum) was collected to contextualise both micronutrient and mood evaluations within the peripartum timeline. Additional variables encompassed parity (primiparous versus multiparous), socioeconomic status indicators (e.g., income, education), body mass index, history of depression or anxiety, pregnancy complications (e.g., gestational diabetes, preeclampsia), and breastfeeding status—elements recognised to confound the association between nutrition and mood disorders.

The third domain pertained to the exposure of interest, specifically micronutrient status. We obtained data regarding the types and quantities of micronutrients assessed (such as Fe, Zn, Mg, Vit D, B12, folate, Se), the biological matrix employed for quantification (including serum, plasma, or RBC), and the analytical techniques utilised (such as ELISA, HPLC, AAS, or chemiluminescence). When evaluating dietary intake instead of biochemical status, the dietary assessment instrument was identified (e.g., FFQ, 24-h recall, 3-day food record), along with details regarding its validation status and nutrient database origin. The timing of micronutrient evaluation (e.g., third trimester, 4 weeks postpartum, 6 months postpartum) was documented to facilitate alignment with PPD outcome measures. The units of concentration, reference ranges, and clinical threshold values utilised to determine deficiency were documented upon reporting.

The fourth domain encompassed all facets of PPD evaluation. The particular instrument employed for assessing depressive symptoms was recorded (e.g., EPDS, CES-D, BDI, PHQ-9), including the total item count, scoring range, and clinical significance cut-off criterion. The timing of administration in relation to childbirth was categorised into early postpartum (≤6 weeks), middle (6–12 weeks), and late (>12 weeks). Various research utilising structured diagnostic interviews based on DSM or ICD were acknowledged. When PPD was analysed as a continuous variable, mean scores and standard deviations were recorded; when analysed categorically, prevalence rates were calculated.

The fifth domain focused on documented outcomes and statistical analyses. The relationship between micronutrient status and PPD was determined (positive, negative, or null), along with statistical measures such as ORs, beta coefficients, RR, 95% CIs, and exact *p*-values. Documentation included specifics regarding the statistical models utilised, specifying whether univariate or multivariate analyses were conducted. In the use of multivariate models, the parameters incorporated (e.g., age, socioeconomic status, parity, body mass index, baseline mental health) were enumerated to assess the sufficiency of confounding control. Subgroup analyses, dose–response analyses, and sensitivity analyses were reported as applicable. Studies doing mediation or interaction analyses, such as examining vitamin D deficiency as a moderator of postpartum depression risk in women with elevated BMI, were identified for further interpretive synthesis.

All data were retrieved utilising structured Excel spreadsheets and cross-validated for accuracy and comprehensiveness. The aforementioned variables form the basis for the narrative synthesis and are concisely presented in Table 1, which provides a thorough overview of the characteristics and key findings of all included studies. These data elements also affect the risk of bias evaluation and facilitate the qualitative interpretation of evidence provided in the Results and Discussion sections.

### 2.8. Assessment of Risk of Bias

A systematic critical evaluation of the Newcastle–Ottawa Scale (NOS) was conducted to thoroughly examine the methodological quality and potential bias risk of the included studies. This methodology aims to assess non-randomized research, including prospective and retrospective cohort studies, case–control studies, and cross-sectional designs, which constituted the principal study categories in this systematic review. The NOS was selected due to its efficacy in observational research and its prevalent application in systematic reviews according to PRISMA and Cochrane Collaboration guidelines.

The NOS evaluates the internal validity of each study across three primary domains: the selection of study groups (up to 4 points), the comparability of groups based on design or analysis (up to 2 points), and the ascertainment of exposure or outcome according to study type (up to 3 points). Each study was assigned a maximum total score of 9, with a higher score indicating a lower likelihood of bias and greater methodological rigour.

In the Selection domain, each study was assessed for the representativeness of the exposed or case–cohort, the methodology utilised for selecting non-exposed or control groups, the reliability of micronutrient exposure assessment (e.g., biochemical measurement of vitamin D, ferritin, folate, or B12), and the documentation verifying that the outcome of interest (i.e., PPD) was absent at the baseline in longitudinal studies. In case–control studies, identical criteria were employed to delineate cases and choose appropriately matched controls.

The Comparability domain assessed whether the studies sufficiently controlled for key confounding variables known to influence the risk of PPD. This included sociodemographic and biological characteristics such as SES, BMI, parity, breastfeeding status, and previous psychiatric history. Points were awarded based on meteorological studies employing statistical adjustments, matching, or stratified analyses to mitigate the influence of these factors.

The Outcome/Exposure domain evaluated the validity and reliability of PPD measurement devices (e.g., EPDS, CES-D) and the timing of assessments in cohort studies. This domain in case–control studies assessed whether exposure to the micronutrient of interest was determined using validated methods, such as standardised blood assays or structured dietary recall instruments, and whether the timing of exposure aligned appropriately with the clinical onset of PPD symptoms.

Two reviewers independently evaluated each of the enumerated studies. In cases of dissent, consensus was reached through dialogue, and a third reviewer was involved where necessary. Subsequently, each study received a cumulative NOS score, which determined the predominant type of bias it was likely to exhibit:

The risk of bias is categorised as low (7–9 points), moderate (5–6 points), or high (<5 points).

Table 1 presents the comprehensive ratings for each study, categorised by NOS domains. This table assists the reader in discerning the methodological advantages and disadvantages of the research, while elucidating the connections between micronutrient deficiencies and postpartum depression with enhanced clarity and assurance. Incorporating this table ensures that the procedures are responsible and substantiates the findings drawn from the narrative synthesis.

### 2.9. Methods of Assembly

A narrative synthesis was employed to integrate the results due to the variations in study design, demographic variables, biomarker timing, and the individual micronutrients assessed. This approach facilitated a methodical, qualitative assessment of outcomes without statistical aggregation, deemed inappropriate due to the heterogeneity of the included studies.

Research was categorised based on the nutrient analysed (e.g., 25[OH]D, Fe, Folate, B12, Zn, Mg), the period of nutritional evaluation (antenatal versus postpartum), and the date and instrument utilised for postpartum depression assessment (e.g., EPDS, CES-D). We examined the strength and consistency of associations between each food group and PPD symptoms, emphasising studies that considered confounding factors such as socioeconomic status, body mass index, parity, breastfeeding status, and mental health history.

The results were categorised by topic, considering both concordant and discordant outcomes. Discrepancies were examined in light of methodological variables, including sample size, biomarker timing, and the extent of statistical adjustment. The initial evaluation of a meta-analysis was obstructed by inconsistencies in exposure definitions, measurement thresholds, and outcome reporting, which prevented quantitative synthesis. Effect sizes were not consolidated. This narrative synthesis method was selected to illustrate patterns and deficiencies in our comprehension of the relationship between micronutrient status and PPD, while also providing a clinically educated assessment of the existing data.

### 2.10. Assessment of Certainty

The GRADE (Grading of Recommendations, Assessment, Development, and Evaluations) methodology was employed to evaluate the overall certainty of the evidence. However, the lack of a meta-analysis, attributable to discrepancies in study designs, outcome measurements, and exposure definitions, makes a formal GRADE grading impractical. A narrative evaluation of the certainty of findings was performed, taking into account the primary areas typically examined by GRADE: risk of bias, inconsistency, indirectness, imprecision, and publication bias. The majority of the included studies were observational, and while several exhibited a low risk of bias according to the NOS ranking (as seen in Table 1), the observational design inherently limits the strength of causal inference.

Inconsistencies were observed in several nutritional categories, particularly in research examining folate, B12, and 25[OH]D, where the results varied in magnitude and direction. This gap was likely attributable to variations in the timing of nutrition and PPD assessments, population characteristics, and unmeasured factors. A few investigations utilising surrogate time or biomarkers demonstrated indirectness, either inadequately reflecting perinatal nutritional status or the true beginning of PPD. Imprecision was a notable concern in smaller studies, particularly those with fewer than 100 participants, marked by wide confidence intervals (when provided) and unreliable estimates. Although publication bias could not be directly assessed due to the absence of quantitative pooling, the frequency of positive associations in certain nutrient domains (e.g., 25[OH]D and PPD) indicates the possibility of selective reporting.

In conclusion, numerous research included exhibit sound methodology, yet the overall certainty of the data remains modest. This is mostly due to the observational nature of the data, significant clinical heterogeneity, and the absence of standardised outcome impact estimates. These constraints highlight the imperative for comprehensive, longitudinal research and randomised nutritional interventions throughout the postpartum phase to strengthen the current evidence base.

## 3. Results

The initial database search identified 3486 records across PubMed, Scopus, and Web of Science as of April 2025. Upon eliminating duplicates, 2712 unique articles remained. Subsequently, they were evaluated based on title and abstract. A total of 117 full-text papers were gathered for an in-depth review. Following comprehensive text evaluation, 98 papers were excluded for the subsequent reasons: ineligible population (*n* = 24), absence of relevant micronutrient data (*n* = 31), absence of a validated PPD assessment (*n* = 28), and inappropriate study design, encompassing narrative reviews or editorials (*n* = 15). Ultimately, 19 publications satisfied the inclusion criteria and were incorporated into the final qualitative synthesis.

The PRISMA 2020 flow diagram (Figure 1) illustrates the entire selection process.

The illustration depicts the progression of records through the various stages of the systematic review. The process entails locating records via database searches and citation tracking, eliminating duplicates and non-compliant entries, screening titles and abstracts, assessing complete texts for eligibility, and ultimately including studies into the qualitative synthesis. At every stage, there are justifications for excluding certain elements. The analysis ultimately comprised 19 studies examining the correlation between vitamin deficits and postpartum depression, as it is described in Table 2.

### 3.1. Vitamin D (25-Hydroxyvitamin D)

Vitamin D, particularly blood 25-hydroxyvitamin D [25(OH)D], was the most extensively researched micronutrient concerning PPD, with 13 out of 19 studies examining this biomarker. Nine of the studies employed prospective cohort designs, three utilized case–control designs, and one was a cross-sectional study. The timing of 25(OH)D level measurements varied significantly throughout the included trials. The period encompassed the second trimester, late pregnancy, immediately post-birth, and up to 6–8 weeks postpartum. This indicates varying perspectives on the timing of vitamin D levels’ impact on mental health during pregnancy and postpartum.

Nine of the thirteen studies identified a statistically significant inverse correlation between 25(OH)D levels and PPD symptoms. Murphy et al. (2010) observed 97 exclusively nursing moms for seven months post-delivery, doing monthly assessments of both 25(OH)D and EPDS scores [81]. A consistent negative correlation was identified between vitamin D levels and depression symptoms [81]. Similarly, Gur et al. (2014) demonstrated that diminished serum 25(OH)D levels during the second trimester were significantly associated with elevated EPDS scores at 1 week, 6 weeks, and 6 months postpartum in a cohort of 208 pregnant women [89]. Pillai et al. (2021) employed a substantial matched case–control design to demonstrate that women with PPD exhibited significantly reduced levels of vitamin D compared to those without PPD at 4–6 weeks postpartum [82].

Furthermore, Accortt et al. (2021) and Fu et al. (2015) provided additional evidence that antenatal vitamin D levels can forecast the risk of PPD. Low levels of 25(OH)D assessed during mid-pregnancy or within the first 48 h postpartum were associated with an increased risk of PPD [86,88]. Chong et al. (2024) and Lamb et al. (2018) similarly saw data consistent with this trend; however, not all time points achieved statistical significance [83,90].

Conversely, four investigations did not identify any significant correlations between vitamin D levels and symptoms of PPD. Noshiro et al. (2023) employed a rigorous longitudinal methodology, including many 25(OH)D assessments during pregnancy and postpartum; nonetheless, they found no statistically significant correlations between vitamin D levels and EPDS scores [77]. Desirée et al. (2024), in a large-scale population-based cohort study (*n* = 2483) utilising the CES-D to assess depression, found no significant associations [87]. Lin et al. (2019) and Yuvaci et al. (2020), both case–control studies with moderate sample sizes, found no differences in vitamin D levels between the PPD and control groups [91,92].

Diverse findings may result from various methodological and contextual factors. The reliability and comparability of the results may have been influenced by variations in the methodologies employed to measure 25(OH)D, disparate thresholds for vitamin D deficiency, seasonal timing of blood sample collection, geographical location of study populations, and insufficient control for confounding variables such as socioeconomic status, body mass index, parity, sun exposure, breastfeeding practices, and mental health history. Certain studies examined 25(OH)D levels at a singular point in time, perhaps overlooking fluctuations in vitamin D levels over the peripartum period.

Despite many anomalies in the data, the comprehensive evidence indicates a negative correlation between low maternal vitamin D levels and symptoms of postpartum depression. This indicates that insufficient vitamin D levels could be a changeable biological risk factor. The quality of the evidence is diminished due to reliance on observations, potential residual confounding, and the absence of interventional data. Additional randomised controlled trials are required to elucidate the causal association and determine whether vitamin D supplementation may aid in the prevention of postpartum depression.

### 3.2. Cobalamin and Folic Acid

Folate (B9) and vitamin B12 are crucial for one-carbon metabolism and neurotransmitter synthesis, both of which are linked to the aetiology of PPD. Interruption of these pathways may result in alterations in monoamine synthesis, elevated homocysteine levels, and neuroinflammation, all of which are biological processes intricately associated with mood disorders. Four studies from the previous dataset examined the relationship between folate and/or B12 and PPD. The results were inconclusive, potentially due to inconsistencies in the research design, timing of assessments, and features of the analysed populations.

The study by Abou-Saleh et al. (1999) employed a case–control design to examine serum levels of folate and B12 in three cohorts: healthy postpartum women, women exhibiting depressive symptoms post-delivery, and pregnant women [79]. The findings indicated that individuals with PPD exhibited significantly reduced levels of folate and, unexpectedly, elevated levels of B12. The investigators hypothesised that elevated B12 levels would indicate reactive supplementation, alterations in hepatic function, or modifications in serum distribution postnatally. Despite the limited sample size and lack of dietary control, this study is significant as it was among the first to examine biochemical profiles throughout the peripartum period [79].

Conversely, Chong et al. (2014) conducted substantial prospective cohort research involving 709 people [83]. Serum folate and B12 levels were assessed at 26–28 weeks of gestation, and the EPDS was employed to evaluate PPD three months postpartum. No statistically significant correlations were identified. The null results may have been influenced by the absence of repeated assessments, lack of dietary or genetic stratification (such as MTHFR), and the possibility of misclassification of subclinical deficits [83].

Dhiman et al. (2021) employed a cross-sectional design involving 434 participants to examine serum B12 levels and EPDS scores at six weeks postpartum [60]. Research indicated that women with postpartum depression exhibited significantly reduced levels of B12. The study excluded individuals with preexisting psychiatric or endocrine disorders, hence enhancing the reliability of the data. These findings substantiate the notion that insufficient B12 levels postpartum may influence mood; however, causation remains indeterminate [60].

Blunden et al. (2012) examined folate levels during the first trimester in a substantial cohort (*n* = 2856) and assessed for PPD six months postpartum [80]. No substantial correlation was identified. Some issues were the exclusive use of a singular early pregnancy measurement, the omission of supplementing considerations, and the failure to examine intermediary biomarkers such as homocysteine. The extended duration between nutritional assessments and PPD evaluations may have further diminished the correlations [80].

These studies demonstrate significant variability in the timing of exposure and the subsequent analysis of the subject matter. The varying timings of biomarker collection across the first, second, or third trimester, or postnatally; the disparate definitions of thresholds; and the inconsistent control for confounding factors such as socioeconomic status, body mass index, parity, and breastfeeding status likely contributed to the divergent outcomes. Two studies (Abou-Saleh, Dhiman) established significant associations between folate or B12 and PPD [60,79], but two others (Chong, Blunden) did not. This indicates that subsequent studies must employ identical methodologies [80,83].

Although the outcomes may vary, there is a compelling biological rationale to assert that folate and B12 can influence mood. Their influence on monoaminergic signaling, neurotrophic support, and inflammatory balance elucidates their mechanisms of action. Future research should investigate the long-term monitoring of nutrients, functional markers such as methylmalonic acid and homocysteine, and gene–nutrient interactions. Conducting RCTs to investigate perinatal supplements and their potential in preventing PPD is logical.

### 3.3. Magnesium and Zinc

Zn and Mg are biologically significant elements that perform many functions in signalling within the CNS, modulating the neuroendocrine system, and altering the immunological system. Both have been associated with the neurobiology of MDD and, more recently, with PPD. Evidence suggests that their absence may result in alterations in NMDA-R function, diminished BDNF synthesis, dysregulated HPA responses, and increased neuroinflammation. These systems are particularly crucial during the postpartum period, when hormonal fluctuations occur, the immune system adjusts, and individuals are more susceptible to mood disorders.

This review examines two PC studies that investigated the relationship between Zn and/or Mg and PPD symptoms. The outcomes for Zn were analogous; however, the findings for Mg were ambiguous. Wojcik et al. (2006) conducted the initial investigation examining blood zinc and magnesium levels in a cohort of 66 women throughout the third trimester and at 3 and 30 days postpartum [84]. EPDS was administered consistently at each time point. The research indicated that women exhibiting elevated EPDS scores demonstrated significantly reduced Zn levels at both 3 and 30 days postpartum. This indicates a sustained association between low zinc levels and depressive symptoms during the early postpartum phase. Conversely, no substantial correlation existed between Mg levels and EPDS scores at any time interval [84]. The longitudinal nature of this investigation, along with the periodic biochemical and psychological assessments, enabled inferences regarding micronutrient status and symptom progression over time. The conclusions are weak because to the limited sample size and the failure to consider potential confounders such as socioeconomic status, body mass index, C-reactive protein, parity, or supplementation status.

In a PC study, Roomruangwong et al. (2016) examined zinc levels after T3 and 4–6 weeks postpartum, contrasting women with elevated and diminished EPDS scores [85]. The high-EPDS group had significantly reduced levels of Zn, corroborating the findings of Wojcik et al. [84]. This study provided additional data supporting the notion that insufficient zinc during pregnancy or shortly postpartum may contribute to symptoms of PPD. The internal validity was enhanced by stringent exclusion criteria for medical or obstetric conditions, notwithstanding the absence of measurements for nutritional intake and inflammatory markers. This study did not examine magnesium.

Zn is recognised for modulating IL-6 and TNF-α levels, maintaining hippocampal plasticity, and facilitating GABAergic transmission. A deficiency of Zn may also diminish the synthesis of 5-HT and impair the body’s defences against oxidative damage in regions of the CNS that are susceptible to emotional stress. Due to physiological losses, insufficient food intake, or heightened demands from the foetus, early postpartum depletion may raise the likelihood of mood disorders, particularly in the absence of reserves to compensate. Neither study established a definitive connection between Mg and PPD, despite its equal significance in neuromodulation and HPA regulation [94]. One reason may be that serum magnesium is not an effective indicator of total body storage, as only around 1% is located in extracellular compartments. Tests assessing intracellular or erythrocyte-based magnesium levels may more accurately reflect the body’s functional status. Neither study examined chronic stress, sleep deprivation, or other factors that may influence magnesium metabolism.

The existing evidence indicates a consistent, albeit preliminary, correlation between low zinc levels and an increased risk of PPD. Both investigations identified this correlation multiple times throughout the PP period and employed validated screening methodologies. The inadequate sample sizes, lack of control for confounding variables, and incomplete nutritional profiling—such as missing data on intake, inflammatory biomarkers, and prenatal vitamin usage—remain significant issues. Conversely, the effects of Mg remain unclear due to insufficient data, potential issues with biomarker selection, and the timing of assessments.

Given the mechanistic rationale and the prevalence of subclinical deficiencies in zinc and magnesium among pregnant women, particularly in low- and middle-income countries and high-stress environments, future research should employ prospective, adequately powered designs with repeated biomarker evaluations throughout the second and third trimesters, as well as multiple postpartum intervals. Factors such as BMI, SES, parity, breastfeeding status, CRP, and multivitamin usage should be incorporated into multivariate models. Furthermore, investigations examining the interactions among nutrients (such as the equilibrium of zinc and copper or the ratios of magnesium and calcium) and their influence on neuroendocrine and inflammatory parameters may enhance our comprehension of how micronutrients impact the pathophysiology of PPD more comprehensively.

It is important to note that the primary emphasis of this systematic review was the association between micronutrients and PPD; however, the studies included often referenced the broader multifactorial context of the disorder. Common causes of PPD encompass psychological, immunological, hormonal, and lifestyle factors, which may interact with micronutrient status to affect vulnerability and clinical outcomes. Nonetheless, a formal quantitative or qualitative synthesis was unattainable within the confines of the current review due to the inconsistent and unmethodical evaluation of these variables across the eligible studies. However, their inclusion here underscores the possibility that micronutrient deficiencies may interact with these additional pathways rather than functioning in isolation. Future research aimed at evaluating the combined influences will be necessary to elucidate the integrative mechanisms underlying PPD.

Table 3 provides a short summary of the evidence for each micronutrient, such as the number of studies included, the direction and consistency of the associations, and important limitations.

Table 3 shows how many studies have looked at each micronutrient, the direction of the link between the micronutrient and PPD that has been seen, the overall consistency of the evidence, and the main methodological problems.

## 4. Discussion

### 4.1. Summary of the Results

This systematic review meticulously evaluated the relationship between vitamin inadequacies and PPD across 19 observational studies, encompassing various geographical settings, methodological approaches, and biochemical assessments. The data suggest that specific micronutrients may considerably affect susceptibility to PPD, although the strength and consistency of these associations differed by nutrient, research methodology, and time of evaluation.

The ingredient most extensively studied was 25[OH]D, assessed in 13 investigations employing both cohort and case–control methodologies. Nine studies demonstrated a statistically significant inverse association between 25[OH]D levels and the severity of PPD symptoms, as measured by the EPDS or the Center for Epidemiologic Studies Depression Scale (CES-D), particularly when 25[OH]D was examined during the second or third trimester. The results suggest a potential gestational period during which vitamin D insufficiency may affect neuropsychiatric outcomes that persist into the postpartum phase. Nonetheless, several studies did not demonstrate significant correlations, and diversity in timing, deficient criteria, and adjustments for confounding variables (e.g., BMI, SES, parity) limit the comparability of findings. Folate and B12 were analysed in four studies, resulting in equivocal findings. A study demonstrated a negative link between folate and PPD, whereas another revealed unexpectedly elevated B12 levels in individuals with PPD. Two further comprehensive cohort studies demonstrated no significant association between nutrients and depressive symptoms throughout the postpartum period. These discrepancies may suggest differences in assessment timeliness, underlying nutritional status, or unconsidered confounding variables such as supplementation methods, dietary quality, or inflammation.

Only one study assessed ferritin as a marker of iron status. This study demonstrated a robust and statistically significant inverse correlation between ferritin levels and PPD, suggesting that even mild iron shortage may increase vulnerability to mood disorders following childbirth. Despite their convincing nature, these findings require replication across several cohorts and adjustment for inflammatory markers, such as CRP, which may influence the interpretation of ferritin levels. Zn and Mg were assessed in two distinct studies. Both trials evaluating Zn demonstrated significantly reduced levels in women with elevated EPDS scores, thus supporting a consistent correlation between Zn deficiency and depressive symptoms throughout the postpartum phase. Magnesium, however, did not demonstrate a statistically significant association with periodontal disease in the available data; still, limits in biomarker sensitivity, such as reliance on blood magnesium rather than intracellular measures, may partially clarify these null findings.

### 4.2. Comprehension in Relation to Prior Research

The findings of this study mostly align with an expanding corpus of research indicating that specific micronutrient deficiencies may contribute to the pathophysiology of PPD. 25-hydroxyvitamin D (25[OH]D) was the most extensively studied micronutrient and had the most significant correlation with depressive symptoms in the postpartum period. Nine of the thirteen studies in this review examining vitamin D levels identified a statistically significant inverse correlation between serum 25[OH]D levels and EPDS scores. These findings align with those of other extensive cohort studies and meta-analyses that have identified insufficient vitamin D as a significant risk factor for PPD. Wang et al.’s meta-analysis indicated that diminished vitamin D levels were associated with an increased incidence of depression both prenatally and postnatally (OR = 3.67; 95% CI: 1.72–7.85). This parallels the direction and magnitude of the associations identified in the papers we analysed [95].

The enduring strength of this association is evidenced by its presence in several regions globally (such as Turkey, China, and the United States) and across distinct temporal stages (including the second trimester, early postpartum, and six months postpartum). Our research indicates that investigations by Gur et al., Fu et al., Lamb et al., and Accortt et al. demonstrated a significant correlation between low levels of 25[OH]D measured during the second or third trimester or immediately postpartum and elevated EPDS scores. The findings indicate that vitamin D influences the nervous system in pregnant women and new mothers via several biological mechanisms, including the modulation of pro-inflammatory cytokines, alteration of serotonin synthesis, and regulation of the HPA axis activity [88,89,90].

Two high-quality prospective cohort studies examined Zn and identified a significant negative correlation with PPD symptoms in both cases. These findings align with existing psychiatric research regarding zinc’s involvement in mood regulation, believed to occur via NMDA receptor antagonism, enhancement of brain-derived neurotrophic factor (BDNF) activity, and antioxidant properties. Our assessment included two research, Wojcik et al. and Roomruangwong et al., and their concordant conclusions lend credence to the notion that zinc plays a significant role in the biology of PPD. Both investigations examined zinc levels in the late third trimester and/or immediately postpartum, indicating that diminished levels during this period may significantly impact mood regulation [84,85].

The role of Mg remains undefined. Wojcik et al. conducted the sole investigation of magnesium levels and found no significant correlation with EPDS scores. This outcome contrasts with previous studies that identified a correlation between low magnesium levels and mood disorders in the general population [84]. Our review did not identify any correlations, potentially due to issues with the methodologies employed. For instance, we solely examined serum magnesium levels, which do not accurately reflect total body reserves and intracellular concentrations—the biologically active magnesium pool. The limited sample size and brief follow-up duration post-delivery may have hindered the identification of a meaningful correlation.

The research conducted by Albacar et al. identified a strong and statistically significant correlation between ferritin levels and PPD [76]. This aligns with our understanding of iron’s function in dopamine synthesis, mitochondrial activity, and brain modification. This study included repeated assessments postpartum (at 48 h, 8 weeks, and 32 weeks), revealing a persistent association between low iron levels and elevated EPDS scores. These findings corroborate previous studies indicating that administering iron to postpartum mothers with iron deficiency helps alleviate fatigue and sadness.

The outcomes for folate and vitamin B12 were ambiguous. Abou-Saleh et al., Chong et al., and Dhiman et al. conducted studies that yielded divergent outcomes. Some identified robust correlations, but others found none [60,79,83]. Abou-Saleh et al. demonstrated that women with postpartum depression exhibited reduced folate and elevated B12 levels [79]. Chong et al. and Blunden et al. did not identify any substantial correlations. The discrepancies indicate significant uncertainty in the psychiatric literature on the impact of one-carbon metabolism on depression. Potential explanations include discrepancies in supplement use, dietary variations, unaccounted genetic variants (such as MTHFR), and insufficient control for additional factors such as socioeconomic status, nutrition, and concurrent health issues [80,83].

The findings of this research indicate that micronutrient levels, particularly 25[OH]D, Zn, and ferritin, may constitute a modifiable risk factor for PPD. The divergent results for folate, B12, and magnesium indicate the necessity for additional, more rigorous research employing consistent methodologies and simultaneous assessment of nutrients and outcomes. The interactions among these nutrients, as well as their relationship with psychosocial and hormonal stressors specific to the perinatal period, require further investigation to elucidate causative factors and potential synergistic effects.

### 4.3. Biological Mechanisms and Pathophysiological Validity

Our systematic study identified robust biological correlations between several micronutrient deficiencies and PPD. The causes encompass neuroimmune regulation, neurotransmitter synthesis, oxidative equilibrium, and neuroendocrine homeostasis. These systems are particularly crucial during the postpartum period, characterised by rapid hormonal fluctuations, heightened physiological demands, and an increased susceptibility to mental health issues.

Thirteen of the nineteen studies examined vitamin D, the most extensively researched nutrient. Numerous investigations, including those conducted by Fu et al., Gur et al., and Murphy et al., identified a significant negative correlation between blood 25-hydroxyvitamin D [25(OH)D] levels and PPD symptoms, as assessed by the EPDS. VDRs are located in many regions of the brain that regulate mood, including the prefrontal cortex and the hippocampus. The active form of vitamin D enhances the expression of TPH2, which regulates serotonin synthesis, and modifies the release of neurotrophic factors such as BDNF. It also reduces the concentrations of pro-inflammatory cytokines such as IL-6 and TNF-α, which are elevated in those experiencing depression. Insufficient vitamin D intake during pregnancy or postpartum may disrupt the equilibrium of neurochemicals and neuroimmune cells, potentially increasing the likelihood of depressive symptoms [81,89].

Albacar et al. investigated iron levels as serum ferritin, which were significantly associated with PPD. Ferritin levels assessed within 48 h postpartum may forecast depressive symptoms at 8 and 32 weeks. Iron serves as a cofactor for enzymes essential in the synthesis of dopamine, norepinephrine, and serotonin, which are crucial neurotransmitters that regulate mood. Insufficient iron intake can disrupt neurotransmission and diminish mitochondrial energy production. Both factors are crucial for maintaining emotional resilience during the physically challenging postpartum period [76].

Two investigations, conducted by Wójcik et al. and Roomruangwong et al., examined zinc, and both identified significant negative correlations between blood zinc levels and PPD scores. Zinc alters synaptic function, inhibits NMDA receptors, and facilitates BDNF expression. It additionally safeguards cells from harm and regulates immune system functionality. During periods of significant stress, such as postpartum recovery, serum zinc levels may decrease due to hepatic storage. This exacerbates the impairment and significantly impacts neuropsychiatric outcomes [84,85].

Four studies examined folate and vitamin B_12_; nevertheless, their findings were inconsistent. Abou-Saleh et al. discovered that women with postpartum depression exhibited significantly reduced folate and elevated B_12_ levels [79]. Nevertheless, other studies, including that of Chong et al., did not identify any statistically significant alterations. Both vitamins remain essential for one-carbon metabolism, which produces S-adenosylmethionine (SAMe), a methyl donor necessary for neurotransmitter metabolism, DNA repair, and the integrity of membrane phospholipids. Issues with these systems, particularly elevated homocysteine levels resulting from folate/B_12_ metabolism dysfunction, might impair neuronal function and complicate mood regulation [83].

This review did not include any trials on selenium; nonetheless, other research indicates its potential significance in mood regulation postpartum. Selenium is crucial for the function of glutathione peroxidase and other selenoenzymes that safeguard cells from oxidative stress. It also facilitates the metabolism of thyroid hormones, influencing mood, cognition, and energy equilibrium. Future studies on the impact of nutrition on PPD should consider selenium, as it may significantly influence underlying mechanisms.

Wójcik et al. examined magnesium and determined that it was not significantly associated with PPD in their cohort. There is substantial data indicating that magnesium can assist in regulating mood. Magnesium influences the HPA axis, functions as a natural NMDA receptor antagonist, and alters neuronal excitability. It is essential to note that blood magnesium levels are not an accurate indicator of the total magnesium content in the body. Subsequent study may benefit from assessing intracellular magnesium levels [84].

In summary, our findings indicate that deficiencies in vitamin D, iron, and zinc are most consistently associated with an elevated risk of postpartum depression, supported by robust mechanistic evidence. These nutrients influence various cerebral functions, including neurotransmitter synthesis, neurotrophic signalling, mitochondrial energy production, and immune system regulation. While folate and vitamin B_12_ appear to be significant for physiological functions, the inconsistency of data indicates the necessity for standardised methodologies and prolonged study durations. Biological evidence advocates for the incorporation of micronutrient screening in prenatal and postpartum care, substantiating the exploration of nutritional therapy as a component of a comprehensive strategy to prevent and address postpartum depression.

### 4.4. Advantages and Disadvantages

This systematic review provides a comprehensive and methodologically rigorous examination of the association between micronutrient deficiencies and PPD, a complex and multifaceted mental health disorder that remains inadequately acknowledged and addressed. This study adheres to the PRISMA 2020 principles and documents a complete methodology in PROSPERO prior to data extraction (CRD420251028514). This ensures the reproducibility of data and diminishes the likelihood of selective reporting, so providing a robust foundation for evidence-based conclusions.

The search methodology employed across three principal electronic databases (PubMed, Scopus, and Web of Science), using backward citation analysis, was comprehensive and intended to mitigate publication bias. Incorporating literature up to April 2025 enabled the utilisation of the most recent evidence in the domain. The eligibility conditions were carefully articulated. Only studies employing objective biochemical assessments of micronutrients (such as serum 25[OH]D, ferritin, B12, folate, and zinc) and validated screening instruments for postpartum depression (mostly the EPDS, along with the CES-D) were included. This enhanced the validity of the investigations and facilitated comparative analysis.

The application of the NOS to assess bias risk across all studies constituted a significant methodological advantage. The tool enabled a thorough assessment of selection methods, confounding control, and result verification, facilitating the categorisation of research into low, moderate, or high risk of bias. The majority of the studies examined were prospective cohort studies. Such investigations are particularly effective in demonstrating the temporal variations in nutritional levels and their correlation with the onset of depressive symptoms. They are also less susceptible to reverse causality than cross-sectional research.

The research was conducted throughout North America, Europe, Asia, and Oceania, enhancing the external validity of the findings. This variability facilitates the application of conclusions across diverse populations, healthcare environments, and nutritional backgrounds. Nonetheless, it also resulted in increased variability in exposure levels and evaluation time points.

Nonetheless, there are specific issues with the conclusions that complicate their comprehension and applicability to other contexts. The primary issue was the significant variability in methodologies employed throughout the studies, particularly regarding the timing of nutrient assessments (e.g., early versus late pregnancy versus postpartum), the timing and frequency of postpartum depression evaluations (ranging from 48 h to 6 months postpartum), and the determination of cutoff values for the EPDS. This rendered a meta-analysis unfeasible. Narrative synthesis facilitated a more refined qualitative interpretation, yet it lacked the statistical capability to quantify impact sizes or assess publication bias.

A significant issue was that the studies employed various methods to account for confounders. Certain research meticulously accounted for significant variables such as socioeconomic status, body mass index, parity, breastfeeding status, marital stability, and a history of mental illness; however, others either failed to employ multivariable models or utilised convenience samples that had external applicability. This contradiction increases the likelihood of residual confounding, thereby undermining the robustness of causal inference. Additionally, several studies excluded women with pre-existing psychiatric illnesses, thus introducing a selection bias that diminishes the applicability of the results for high-risk populations.

A further issue is that the majority of research examines only a single micronutrient at a time, complicating the ability to discern the broader context. The interconnections of nutrients and the influence of overall dietary patterns, gut microbiota, and inflammatory conditions have been infrequently examined; however, they are increasingly recognised as significant determinants impacting nutritional bioavailability and neuropsychiatric outcomes. Serum markers such as zinc and magnesium, frequently utilised despite their limited sensitivity to intracellular state, may insufficiently reflect the availability of these nutrients for utilisation in the brain.

Additionally, discrepancies in laboratory testing, the absence of standardisation in reference ranges, or incomplete data regarding the administration of prenatal vitamins may have introduced measurement bias. The EPDS has been evaluated in multiple languages and is extensively utilised; however, the existence of varying scoring thresholds and the absence of clinical interviews for formal diagnosis (such as the DSM-5 criteria) complicate the proper classification of depression and may have led to misclassification bias.

Finally, PPD is a biopsychosocial disorder, rendering single-factor explanatory models ineffective. Nutritional status is a modifiable component that is technically attainable; however, it is influenced by several social, psychological, genetic, and hormonal determinants. Distinguishing its distinct contribution is challenging, underscoring the necessity for future research utilising integrative, longitudinal, and systems-biology methodologies.

This comprehensive review, despite its limitations, contributes significantly by synthesising current research, identifying consistent associations among specific micronutrients such as vitamin D, iron, and zinc, and establishing a foundation for targeted interventions. This underscores the necessity for future study designs to exhibit greater consistency through standardised biomarker assessments, simultaneous measurements, an expanded array of psychological and social factors, and potentially exploring nutrient-based prevention or adjunctive therapy strategies for at-risk postpartum populations.

### 4.5. Implications for Patients and Subsequent Actions

This systematic study indicates that an increasing number of individuals recognise the significance of maternal nutrition for both physical and mental health throughout the postpartum period. The correlation between insufficient levels of essential micronutrients, particularly vitamin D (25[OH]D), ferritin, and zinc, along with weaker influences from folate and vitamin B_12_, and an increased risk of postpartum depression PPD carries significant clinical ramifications for prevention, screening, and therapy.

Incorporating micronutrient screening into standard prenatal care is a pragmatic and efficacious method to prevent complications. Dietary guidance, fortified foods, and targeted supplementation are several straightforward and economical methods to address numerous identified deficiencies. The early stages of pregnancy, particularly during the late second and early third trimesters, appear to be a critical period when micronutrient levels may influence the brain’s susceptibility to PPD. This may occur due to alterations in the control of the HPA axis, neuroinflammation, and monoamine neurotransmission. Monitoring levels of 25[OH]D, ferritin, and zinc, particularly in women with inadequate diets, low socioeconomic status, darker skin, limited sun exposure, or a history of depression, may assist in identifying individuals at elevated risk for postpartum depression and determining the appropriate timing for preventative interventions.

Current antenatal guidelines often permit women to consume only folic acid and iron supplements. This mostly aids foetal development and reduces the chance of neural tube defects or anaemia. This review indicates the necessity for a more individualised and comprehensive approach to nutrition. Vitamin D has emerged as a significant possibility for screening, as numerous studies indicate that low levels during pregnancy or shortly after birth are strongly associated with elevated scores on the EPDS. Regularly monitoring serum 25[OH]D may benefit both moms and infants in two ways.

In obstetrics and primary care, validated screening tools such as the EPDS are widely employed to assist in the therapeutic management of PPD. Incorporating concurrent biochemical testing could enhance their utility. A woman exhibiting mild depressive symptoms in the early postpartum phase may not immediately meet diagnostic criteria; nonetheless, she could still gain from targeted therapies if low serum 25[OH]D or ferritin levels are identified. This integrated strategy may facilitate the convergence of mental health and nutritional medicine, hence simplifying the implementation of multidisciplinary treatment plans involving obstetricians, psychiatrists, nutritionists, and general practitioners.

Moreover, while numerous health systems predominantly prioritise the well-being of neonates post-delivery, maternal health typically receives diminished attention. Incorporating nutritional assessments into postpartum visits, particularly within the initial six weeks when numerous instances of exhaustion and mood fluctuations associated with micronutrient deficiencies arise, may facilitate the identification of subclinical postpartum depression that could otherwise go undetected. Incorporating nutrients into an individual’s diet, predicated on laboratory-validated deficits, constitutes a comparatively low-risk enhancement to conventional psychological or pharmacological therapies. This may be particularly alluring to moms apprehensive about initiating antidepressant use during nursing.

Nevertheless, existing deficiencies in the current database hinder the translation of these findings into therapeutic advice. There is no consensus on the precise amounts of essential nutrients that elevate the risk of PPD, and insufficient data exists regarding optimal dosing regimens, duration of supplementation, or the efficacy of monotherapy relative to multi-nutrient interventions. The variability in measurement intervals, nutrient assessment methodologies, and approaches to controlling confounding variables in existing research complicates the formulation of robust recommendations at this time.

Future research ought to concentrate on extensive, meticulously designed RCTs examining both the preventive and therapeutic impacts of incorporating micronutrients into individuals’ diets on PPD outcomes. These trials should utilise same methodologies for nutrient detection, implement consistent diagnostic criteria for PPD (ideally aligned with DSM-5), and incorporate long-term follow-up to assess the duration of benefits. Conducting subgroup analyses will be essential to identify the populations that derive the greatest benefit, including women who are undernourished, overweight, have gestational diabetes, or suffer from inflammatory disorders.

Future research should transcend the single-nutrient model and adopt a systems-biology approach that considers the intricate interactions among nutrients, hormones, epigenetic modifications, inflammatory markers, gut microbiota composition, and psychosocial stressors. Recent research indicate that micronutrient levels may influence mood through the gut–brain axis, immunomodulation, and oxidative stress pathways. These pathways are particularly significant during the postpartum phase when hormonal fluctuations occur rapidly.

From a public health and policy perspective, these findings indicate the necessity to enhance maternal nutrition programs, particularly in low- and middle-income countries where mental health issues and nutritional inadequacies are prevalent. Perinatal mental health guidelines must incorporate screening for nutrient deficiencies, and perinatal healthcare practitioners should be educated to identify and address dietary risk factors associated with depression. Incorporating mental health outcomes in future studies of prenatal supplement programs could strengthen the argument for their expansion and enhancement.

This investigation indicates that micronutrient-based therapies may be effective in both preventing and treating PPD. Further research is required to delineate optimal practices; nevertheless, incorporating biochemical screening and nutritional interventions into standard prenatal care appears to be a scalable, non-stigmatizing, and biologically grounded approach to enhancing maternal mental health.

Our findings underscore the critical need of early nutritional screening in prenatal care from a therapeutic perspective. Regular assessment of critical micronutrients such as zinc, vitamin D, and vitamin B12 may assist in identifying women at heightened risk for postpartum depression. Incorporating nutritional counselling or targeted supplements into prenatal and postpartum care protocols may serve as a safe and cost-effective strategy for issue prevention. The standardised adoption of these procedures may improve mother mental health and promote baby development.

Future research should concentrate on rigorously structured longitudinal cohort studies and randomised controlled trials that investigate various micronutrients together to find possible synergistic effects. To improve comparability across studies, the timing of biomarker evaluation and the use of established diagnostic tools for postpartum depression must be standardised. Furthermore, research on gene–nutrient interactions, especially with MTHFR and other polymorphisms affecting micronutrient metabolism, may provide critical insights into individual risk. Ultimately, interventional studies examining the effectiveness, timing, and dose of micronutrient supplementation in preventing postpartum depression are crucial for informing clinical practice and policy.

Consider the potential impact of confounding factors on these findings. The levels of micronutrients and the risk of postpartum depression are affected by nursing behaviours, the season of blood collection, body mass index, parity, socioeconomic position, and prior psychiatric history. The diversity of outcomes across nutrients may be partly due to the lack of full multivariate models, while some research has addressed particular confounders.

It is essential to recognise the limitations of the included research. The majority used observational approaches, marked by often limited sample sizes and susceptibility to residual confounding. The timing of micronutrient evaluations differed considerably across trials, and PPD outcome measures were not uniformly used. The methodological deficiencies undermine the robustness of causal inference and highlight the need for precisely crafted prospective cohorts and randomised controlled trials with stringent control of pertinent factors.

The diversity of biochemical techniques used to assess micronutrients was an additional factor contributing to the discrepancies across the studies. We used several analytical techniques to quantify serum and plasma levels, including atomic absorption spectroscopy (AAS), high-performance liquid chromatography (HPLC), and enzyme-linked immunosorbent assays (ELISA). These methodological changes may complicate direct study comparisons and alter the absolute concentration results. When examining aggregated data, it is crucial to acknowledge this significant shortcoming, despite its prevalence in studies concerning diet and mental health.

The restricted quantity of papers fulfilling the inclusion criteria is a significant limitation of this systematic review. The little data undermines the robustness and generalisability of the findings, requiring care in result interpretation. The ability to reach clear findings is further limited by the variability in research designs and outcome measurements.

## 5. Conclusions

The data concerning folate, iron, magnesium, and selenium is unclear because to methodological inconsistencies and inadequate correction for confounding factors. This comprehensive study demonstrates that deficits in zinc, vitamin D, and vitamin B12 are consistently linked to an increased risk of postpartum depression. These results indicate the prospective benefits of focused nutritional assessment and supplementation in prenatal mental health treatment. Comprehensive, longitudinal, and interventional research is essential to elucidate causation and provide therapeutic recommendations.

Future research should focus on comprehensive, rigorously conducted studies that comprehensively assess the correlation between postpartum depression and nutritional status. Standardised evaluation of micronutrient levels, longitudinal methods, and uniform diagnostic criteria for postpartum depression need specific attention. Such studies are essential for elucidating causal pathways, distinguishing the influence of confounders, and ultimately guiding dietary treatments as curative or preventative strategies.

## Figures and Tables

**Table 1:** Newcastle–Ottawa Scale (NOS) Risk of Bias Assessment for Included Studies.

Author and Year	Selection (Max 4)	Comparability (Max 2)	Outcome/Exposure (Max 3)	Total NOS Score (Max 9)	Risk of Bias
Albacar et al., 2011, [76]	3	2	3	8	Low
Noshiro et al., 2023, [77]	4	2	2	8	Low
Robinson et al., 2014, [78]	3	2	3	8	Low
Abou-Saleh et al., 1999, [79]	2	1	2	5	Moderate
Blunden et al., 2012, [80]	3	2	2	7	Low
Murphy et al., 2010, [81]	3	2	3	8	Low
Pillai et al., 2021, [82]	3	2	3	8	Low
Chong et al., 2014, [83]	3	2	2	7	Low
Dhiman et al., 2021, [60]	3	1	2	6	Moderate
Wojcik et al., 2006, [84]	2	1	2	5	Moderate
Roomruangwong et al., 2017, [85]	3	2	2	7	Low
Accortt et al., 2021, [86]	3	2	3	8	Low
Domacasse et al., 2024, [87]	2	1	2	5	Moderate
Fu et al., 2015, [88]	3	2	3	8	Low
Gur et al., 2014, [89]	4	2	3	9	Low
Lamb et al., 2018, [90]	3	2	3	8	Low
Lin et al., 2019, [91]	2	1	2	5	Moderate
Yuvaci et al., 2020, [92]	2	1	2	5	Moderate
Wang et al., 2023, [93]	3	2	3	8	Low

The Selection domain indicates the representativeness of the study population, the methodology used for exposure assessment (e.g., biochemical measurement of micronutrient levels), and the confirmation that the outcome of interest—postpartum depression—was not present at the study’s outset in longitudinal designs. The Comparability domain evaluates whether studies sufficiently controlled for potential confounding variables, such as socioeconomic status (SES), body mass index (BMI), parity, and baseline mental health status. The Outcome/Exposure domain assesses the use of validated measurement tools for micronutrient assessment and postpartum depression diagnosis (e.g., EPDS, CES-D), as well as the adequacy of scheduling and follow-up procedures. The Newcastle–Ottawa Scale scores categorised the study into three groups: low risk of bias (scores 7 to 9), moderate risk of bias (scores 5 to 6), and high risk of bias (scores below 5).

**Table 2:** Summary of included studies and key characteristics.

#	Author (Year)	Study Design	Micronutrient Focus	Timing of Assessment (Antenatal/Postpartum)	PPD Measure (As Reported)	Main Finding (Direction)	Risk of Bias (NOS)
1	Albacar et al. (2011), [76]	Observational	Vitamin D (grouped in manuscript)	Not specified in text extract	Validated tool (per manuscript)	Not explicitly detailed in text extract	Low
2	Noshiro et al. (2023), [77]	Longitudinal	Vitamin D	Multiple time points antenatal/postpartum	EPDS (per manuscript)	No significant association	Low
3	Robinson et al. (2014), [78]	Observational	Vitamin D (grouped)	Not specified	Validated tool	Not explicitly detailed in text extract	Low
4	Abou-Saleh et al. (1999), [79]	Case–control	Folate, Vitamin B12	Postpartum	Validated tool	↓ Folate; ↑ B12 in PPD group	Moderate
5	Blunden et al. (2012), [80]	Cohort	Folate	1st trimester (antenatal)	PPD at ~6 mo	No significant association	Low
6	Murphy et al. (2010), [81]	Prospective cohort	Vitamin D	Monthly postpartum (exclusively breastfeeding)	EPDS monthly	Inverse association (lower 25(OH)D → higher PPD symptoms)	Low
7	Pillai et al. (2021), [82]	Matched case–control	Vitamin D	4–6 weeks postpartum	Validated tool	Lower 25(OH)D in PPD cases	Low
8	Chong et al. (2014), [83]	Cohort	Folate, Vitamin B12	26–28 weeks gestation	EPDS 3 mo postpartum	No significant association	Low
9	Dhiman et al. (2021), [60]	Cross-sectional	Vitamin B12	~6 weeks postpartum	EPDS	Lower B12 associated with PPD	Moderate
10	Wojcik et al. (2006), [84]	Prospective cohort	Zinc, Magnesium	3rd trimester; 3 & 30 days postpartum	EPDS	↓ Zinc associated with higher EPDS; Magnesium: no assoc.	Moderate
11	Roomruangwong et al. (2017), [85]	Prospective cohort	Zinc	Late pregnancy & 4–6 weeks postpartum	EPDS	Lower zinc in high-EPDS group	Low
12	Accortt et al. (2021), [86]	Observational	Vitamin D	Antenatal	Validated tool	Low antenatal 25(OH)D predicts higher PPD risk	Low
13	Domacasse et al. (2024), [87]	Population-based cohort	Vitamin D	As reported	CES-D	No significant association	Moderate
14	Fu et al. (2015), [88]	Observational	Vitamin D	Within 48 h postpartum	Validated tool	Low 25(OH)D associated with PPD	Low
15	Gur et al. (2014), [89]	Cohort	Vitamin D	2nd trimester → PPD at 1 wk/6 wk/6 mo	EPDS	Inverse association across time points	Low
16	Lamb et al. (2018), [90]	Observational	Vitamin D	As reported	Validated tool	Trend consistent with inverse association (not all time points significant)	Low
17	Lin et al. (2019), [91]	Case–control	Vitamin D	As reported	Validated tool	No significant difference	Moderate
18	Yuvaci et al. (2020), [92]	Case–control	Vitamin D	As reported	Validated tool	No significant difference	Moderate
19	Wang et al. (2023), [93]	Observational	Vitamin D (grouped)	As reported	Validated tool	Inverse association (per manuscript summary)	Low

A summary of the research assessing the correlation between micronutrient status and postpartum depression, organised in accordance with the risk-of-bias table. CES-D: Centre for Epidemiologic Studies; EPDS: Edinburgh Postnatal Depression Scale 25(OH)D: 25-hydroxyvitamin D; Depression Scale.

**Table 3:** Summary of associations between micronutrient deficiencies and postpartum depression (PPD).

Micronutrient	No. of Included Studies	Direction of Association with PPD	Consistency of Evidence	Key Limitations
Vitamin D	11	Majority report inverse association (low 25(OH)D linked to higher PPD risk); some null findings	Moderately consistent	Heterogeneity in timing of blood sampling; limited adjustment for confounders; variability in depression scales
Vitamin B12	2	Mixed: one study lower B12 in PPD, one no association	Inconsistent	Small sample sizes; postpartum-only assessment
Folate	2	One study shows lower folate in PPD; others no association	Inconsistent	Timing of measurement varies; dietary intake vs. serum folate differences
Iron	1	No significant association	Limited	Only one eligible study; small cohort
Zinc	2	Both studies: lower zinc levels linked with higher PPD symptoms	Consistent but limited	Small cohorts; short follow-up
Magnesium	1	No significant association	Limited	Very few data; postpartum-only

## Abbreviations

PPD: Postpartum Depression; PRISMA: Preferred Reporting Items for Systematic Reviews and Meta-Analyses; PROSPERO: International Prospective Register of Systematic Reviews; PICOS: Population, Intervention/Exposure, Comparison, Outcomes, Study design; EPDS: Edinburgh Postnatal Depression Scale; CES-D: Center for Epidemiologic Studies Depression Scale; BDI: Beck Depression Inventory; PHQ-9: Patient Health Questionnaire-9; DSM-5: Diagnostic and Statistical Manual of Mental Disorders, Fifth Edition; ICD-10/11: International Classification of Diseases, 10th/11th Revision; HPA: Hypothalamic–Pituitary–Adrenal (axis); CRH: Corticotropin-Releasing Hormone; TSH: Thyroid-Stimulating Hormone; FT4: Free Thyroxine; PRL: Prolactin; OT: Oxytocin; IL-6: Interleukin-6; IL-1β: Interleukin-1 beta; TNF-α: Tumor Necrosis Factor-alpha; CRP: C-Reactive Protein; 25(OH)D: 25-hydroxyvitamin D; VDR: Vitamin D Receptor; TPH2: Tryptophan Hydroxylase 2; 5-HT: 5-Hydroxytryptamine (serotonin); BDNF: Brain-Derived Neurotrophic Factor; NMDA: N-Methyl-D-Aspartate; MTHFR: Methylenetetrahydrofolate Reductase; SAM: S-Adenosylmethionine; GPx: Glutathione Peroxidase; RBC(s): Red Blood Cell(s); FFQ: Food Frequency Questionnaire; ELISA: Enzyme-Linked Immunosorbent Assay; HPLC: High-Performance Liquid Chromatography; AAS: Atomic Absorption Spectroscopy; OR: Odds Ratio; RR: Relative Risk; CI: Confidence Interval; SD: Standard Deviation; β: Beta coefficient; BMI: Body Mass Index; SES: Socioeconomic Status; RCT: Randomized Controlled Trial; NICU: Neonatal Intensive Care Unit; IUFD: Intrauterine Fetal Demise; AND: Antenatal Depression; ANA: Antenatal Anxiety; MDD: Major Depressive Disorder; GAD: Generalized Anxiety Disorder; BD: Bipolar Disorder; PTSD: Post-Traumatic Stress Disorder.
